# Audio‐guided exercise at home: Preliminary findings from a pilot study on physical activity for people with low vision

**DOI:** 10.1002/pmrj.70081

**Published:** 2026-01-10

**Authors:** Luca Correale, Monica Schmid, Francesco Decortes, Luca Martinis, Giulia Liberali, Luca Bandirali, Gabriella Cusella, Cristina Montomoli

**Affiliations:** ^1^ Human Locomotion Laboratory (LOCOLAB), Department of Public Health, Experimental Medicine and Forensic Sciences University of Pavia Pavia Italy; ^2^ Visual Rehabilitation Center Istituti Clinici Scientifici Maugeri Pavia Italy; ^3^ Movement Analysis Research Section IRCCS Mondino Foundation Pavia Italy; ^4^ Moriggia‐Pelascini Hospital Gravedona ed Uniti Como Italy; ^5^ Human Anatomy Unit, Department Public Health, Experimental and Forensic Medicine University of Pavia Pavia Italy; ^6^ Unit of Biostatistics and Clinical Epidemiology, Department Public Health, Experimental and Forensic Medicine University of Pavia Pavia Italy

## Abstract

**Background**: Individuals with severe visual impairments frequently refrain from regular physical activity due to multiple barriers, including transportation difficulties, financial constraints, and limited availability of exercise programs tailored to their needs. These factors often contribute to a gradual decline in mobility and independence.

**Objective**: To evaluate the feasibility of a self‐administered, audio‐guided training protocol designed to improve mobility in people with severe visual impairments.

**Design**: Nonrandomized controlled clinical trial (number: NCT05956730).

**Participants**: Nineteen adults (55.5 ± 14.8 years, 52% females) with severe low vision or blindness, met the inclusion criteria and completed all the assessments.

**Interventions**: Participants were assigned to an audio‐guided (AUD) group performing a 3‐month home‐based training or a supervised (SUP) group performing identical training in a gym.

**Outcome measures**: Mobility assessment, including Timed Up and Go Low Vision version (TUG‐LV), 1‐minute sit‐to‐stand (1‐MSTS), the 3‐minute step (3‐MSTEP), and Sit and Reach (SR) tests, was conducted every 4 weeks. Physical activity level, satisfaction profile, visual functioning, and caregiver burden were recorded.

**Results**: Participants reported no injuries or physical issues, and adherence was >95%. Both AUD and SUP showed enhancements in TUG‐LV, 1‐MSTS, 3‐MSTEP, and SR (time, *p* <.001), with differences between groups in 1‐MSTS and 3‐MSTEP (time × group, *p* = .04). Physical activity level and satisfaction in physical functioning increased over time (*p* = .002; *p* = .03), with no time per group interaction (*p* = .76; *p* = .37). No significant changes were noted in visual functioning or caregiver burden.

**Conclusions**: Although supervised training showed slightly greater improvements in some outcomes, audio‐guided training is feasible, offering a valuable alternative to enhance mobility when supervision is not viable.

## INTRODUCTION

Low vision is a worldwide health problem, especially in developed countries, due to the increasing older population.^1^ Although the causes of low vision are various ‐such as glaucoma, diabetic retinopathy, and age‐related macular degeneration‐ the associated impairments in visual acuity, visual field, and depth perception can contribute to the mental and physical decline. The resulting disability leads people with low vision to a loss of autonomy and to a decreased quality of life.^2^, ^3^

The physical decline is partially due to functional impairments that reduce walking ability, such as gait and balance issues and muscle weakness of the lower limb.^4^, ^5^ Walking is a fundamental skill for most individual activities and autonomy. Visual impairments and resulting balance issues lead people with low vision to the fear of falling and consequent sedentary behavior.^6^ The result is reduced physical activity and the loss of muscle mass and strength, with worsening of the muscle weakness of the lower limb. In this perspective, exercise is the most obvious and effective intervention for avoiding these issues and reducing functional impairments.^7^

Previous studies demonstrated that programs of supervised resistance training are effective to enhance strength and functional capacities in populations with low vision.^8^ However, most people with visual impairments continue to avoid physical activity due to exercise barriers such as travel and economic issues, as well as the lack of specific exercise programs adapted to visual impairments.^9^ To overcome these barriers and permit people with low vision to exercise safely and effectively at their homes, we hypothesized that resistance training could be prescribed through a series of audio guides. Therefore, we aimed to study the feasibility of an audio‐guided resistance training protocol in a sample of people with low vision or blindness and to test its effect compared to the same supervised training protocol.

## MATERIALS AND METHODS

This nonrandomized controlled trial study was conducted at the Visual Rehabilitation Center, ICS Maugeri in Pavia, Italy. It was approved by the local Ethical Committee (protocol number: 2422) and registered on clinicaltrials.gov (clinical trial number: NCT05956730), and it is in accordance with the Declaration of Helsinki. Prior to enrolling in the present study, each participant signed a detailed informed consent. The document was signed in the presence of his/her caregiver or legal representative to guarantee transparency.

### Participants

The sample size for this pilot study was determined based on methodological recommendations for feasibility studies, which suggest that a sample of 12–30 participants is adequate to assess procedures, acceptability, and preliminary outcomes.^10^, ^11^ Therefore, we aimed to recruit about 20 participants to evaluate the feasibility and acceptability of an audio‐guided home exercise program for individuals with low vision.

In the present study, 23 voluntary people with low vision or blindness were enrolled. The group allocation was based on whether their caregiver could accompany them to the gym. They were then assigned to two groups: audio‐guided training (AUD) or supervised training (SUP). Nineteen participants completed the program and the follow‐up. The inclusion criteria were as follows: adults (age ≥ 18 years), low vision or blind (visual acuity <1/10), binocular visual field residual <30%, and have not taken part in any structured exercise program in the previous 3 months. A comprehensive clinical history was collected to identify any incompatible pathologies with the safe execution of the exercise protocol, thus serving as an exclusion criterion.

### Procedures

Following the enrollment and group assignment, each participant underwent a first visit to complete all physical tests and questionnaires. In addition, participants in AUD group underwent a second visit to receive the audio track and to familiarize themselves with the exercises in the presence of a trainer. The week following the visits, each participant started the exercise protocol.

The exercise protocol was designed based on the most recent World Health Organization guidelines on physical activity.^12^ Considering the lack of low‐vision population‐specific recommendations and to make the home‐based approach safe and simple, tailored modifications were implemented through a collaborative decision‐making process involving both medical professionals and trainers. The AUD group performed a concurrent training (resistance and aerobic) twice a week at home for 12 weeks, listening to the audio track provided by the trainer. Audio instructions covered the steps to perform the training safely and were updated every 4 weeks with load increments. To collect information about adherence to protocol, weekly telephone calls were made to monitor the completion of the sessions. Meanwhile, the SUP group performed the same training protocol in the gym under the supervision of the trainer (for more information about the training, see Supplement 1A). For each participant, the motor function, level of physical activity, quality of life, visual functioning, and caregiver burden were tested before the start, and every 4 weeks until the end of the protocol (during weeks 5, 9, and 13).

### Measures

#### Adherence

The adherence to the training protocol was considered as the percentage of completed training sessions compared to the total number of sessions (*n* = 24) required by the protocol.

#### Motor function

The motor function was assessed with the following tests: Timed Up and Go Test‐Low Vision version (TUG‐LV), 1‐minute sit to stand test (1‐MSTS), the 3‐minute step test (3‐MSTEP) and Sit and Reach test (SR).

The TUG‐LV test is a cost‐effective and reliable test to evaluate the overall functional mobility of people with physical limitations and/or visual impairments.^13^, ^14^ During the TUG‐LV test, the participant is required to get up from a chair, walk the length of a 3‐m path following the voice of the researcher until the turning point, turn around 180°, walk back, and sit again.^15^ The result of the test is the time between the “start” command and the sitting.

The 1MSTS test is used for the assessment of exercise capacity, reflecting the contribution of the lower limb strength. Starting from a seated position on an armless chair (∼50 cm high) with hands positioned at the chest, the participants were instructed to stand up and sit down as quickly as possible for the maximum number of repetitions within 1 minute.^16^, ^17^

The 3‐MSTEP is a physical test that is used to assess aerobic fitness. It is performed using a 15‐cm high step, from which the participant is asked to ascend and descend as many times as possible in 3 minutes.^18^

The SR test is used to evaluate the flexibility of the posterior kinetic chain. The participant, seated with lower limbs stretched out on the ground, barefoot resting on a specific graduated cube, is asked to bend the torso forward and try to reach the furthest point with the fingers. The best value is recorded after having the patient perform three tests spaced with 1 minute of recovery.^19^

#### Level of physical activity

The amount of physical activity prior and during the intervention was recorded through the International Physical Activity Questionnaire Short Form (Italian version).^20^ This questionnaire was used to measure the level of physical activity and the amount of sedentary time through questions about the activities performed in the 7 days prior to the compilation. The results are presented in this study in the form of metabolic equivalent of task (MET) × minute × day.

#### Quality of life

Quality of life was assessed by the satisfaction profile, a questionnaire aimed to measure the level of personal satisfaction of patients on 32 aspects of daily living. It consists of 32 questions (one per aspect), and it is possible to summarize them into five factors (psychological functioning, physical functioning, work, sleep and eating and leisure, and social functioning), of which those related to psychological and physical functioning were considered in the study.^21^

#### Visual functioning

Visual functioning refers to an individual's ability to use their vision in everyday life, encompassing a range of visual abilities (such as visual acuity, visual field, depth perception, contrast sensitivity, and adaptation to different lighting conditions) necessary for performing tasks and navigating the environment. Assessing visual functioning is important because it provides a comprehensive understanding of how vision impairments affect an individual's ability to perform daily activities, maintain independence, and ensure safety (eg, reduced acuity or visual field can impair object recognition, whereas poor depth perception results in difficulty navigating in low‐light environments, increasing the risk of falls and injuries).

To assess visual functioning, we used the National Eye Institute Visual Functioning Questionnaire 25 (VFQ‐25). The VFQ‐25 consists of 25 questions and is divided into 12 subscales, 6 of which were considered for the purposes of the study (general health, near activities, social functioning, mental health, role difficulties, and dependency).^22^

#### Caregiver burden

The level of stress experienced by the person who most cares for the patient with limited autonomy (caregiver), was measured with the Caregiver Burden Inventory. This inventory is a self‐report questionnaire composed of 24 questions and divided into five sections (psychological, objective, physical, social, and emotional stress). In the present study, we considered the total score.^23^

### Statistical analysis

Frequencies were used to describe qualitative variables, with mean and SD to describe quantitative variables. Statistical differences between AUD and SUP groups were tested with Fisher exact test and Mann–Whitney test according to the nature of the variables.

A linear mixed model was used to assess differences in physical and clinical outcomes between groups and over time, including age as a covariate.

The minimal clinically important differences (MCIDs) were estimated for physical tests at baseline using the distribution‐based method.^24^

A *p* < .05 was considered significant. All statistics were performed using STATA software (version 17.0).

## RESULTS

Participants' characteristics are shown in Table 1.

**TABLE 1:** Characteristics of the sample.

	AUD (*N* = 11)	SUP (*N* = 8)	*p* value
Gender (M)	6 (55%)	3 (37.5%)	.65
Age, y	62.6 ± 12.2	45.8 ± 12.7	.01
Height, m	1.67 ± 0.1	1.66 ± 0.1	.96
Body mass, kg	72 ± 15.2	64.1 ± 14.3	.26
Body mass index, kg/m^2^	26 ± 4.5	23.2 ± 4.7	.23

*Note*: Data are frequencies (%) and mean ± SD.

Abbreviations: AUD, audio‐guided training group; SUP, supervised training group.

### Study population

Out of the 23 participants enrolled in the study, 3 completed only the initial assessment, and 1 dropped out of the exercise program in the second week for personal reasons, resulting in a total dropout rate of 17%. Participants in the AUD group reported completing >95% of the workouts without experiencing any injuries or physical issues that prevented them from performing the exercises.

### Physical tests

**FIGURE 1:**
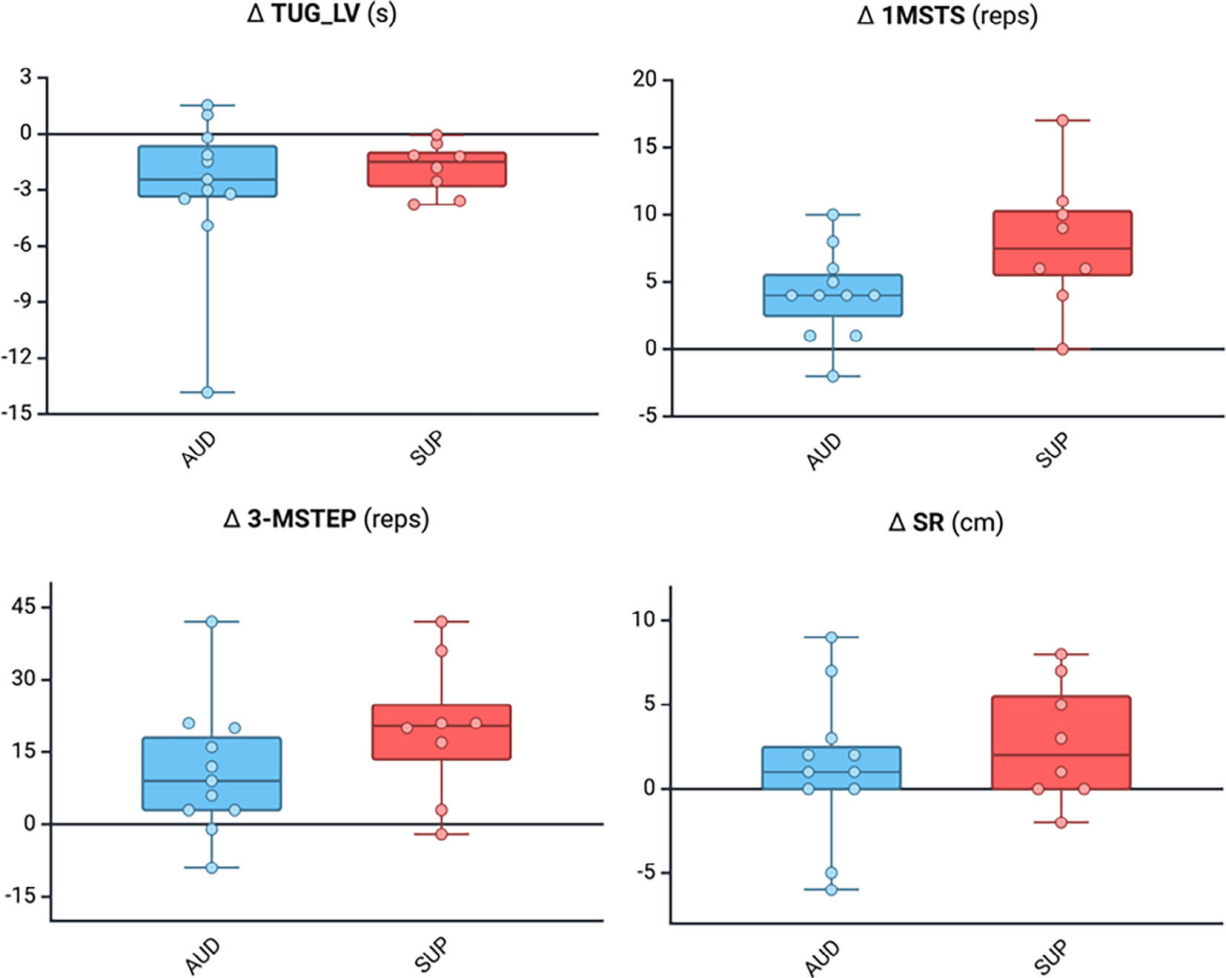
Performance difference between post‐pre intervention. 1‐MSTS, 1‐minute sit to stand test; 3‐MSTEP, 3‐minute step test; AUD, audio‐guided training; SR, sit and reach test; SUP, supervised training; TUG‐LV, Timed Up and Go test‐low vision version; X, mean value.

Table 2 shows the results of physical tests at baseline and during the 3 months of training for AUD and SUP training groups. All physical tests improved significantly after the intervention in both groups, with a significant interaction time per group in the 1‐MSTS and 3‐MSTEP tests (*p* = .003 and *p* = .007, respectively), indicating an improvement over time of the performance. The performance differences between pre‐ and postintervention are more pronounced in these tests (Figure 1).

**TABLE 2:** Changes in motor function during the exercise programs.

	TUG‐LV (s)				
	AUD	SUP	*β*	95% CI	*p* value
Baseline	14.6 ± 3.2	12.8 ± 5.9			
Week 5	11.8 ± 3.4	11 ± 6.1	−2.83	−4.23 to −1.42	<.001
Week 9	11.5 ± 3.1	10.2 ± 6.2	−3.14	−4.54 to −1.73	<.001
Week 13	11.2 ± 2.8	9.9 ± 6.1	−3.44	−4.85 to −2.04	<.001

	1‐MSTS (reps)				
	AUD	SUP	*β*	95% CI	*p*
Baseline	18.7 ± 6.2	21.3 ± 7.3			
Week 5	22.8 ± 5.5	29.1 ± 10.4	4.09	1.74 to 6.43	.001
Week 9	24.7 ± 5.7	29.3 ± 9.8	6	3.65 to 8.34	<.001
Week 13	24.5 ± 4.9	32.5 ± 12.2	5.72	3.38 to 8.07	<.001

	3‐MSTEP (reps)				
	AUD	SUP	*β*	95% CI	*p*
Baseline	56.5 ± 17	64.4 ± 21.7			
Week 5	67.5 ± 15.1	84.1 ± 26.7	11.09	4.79 to 17.38	.001
Week 9	69.7 ± 17.4	91.1 ± 28.1	13.27	6.97 to 19.56	<.001
Week 13	73.3 ± 13.1	94.6 ± 28.2	16.81	10.52 to 23.11	<.001

	SR (cm)				
	AUD	SUP	*β*	95% CI	*p*
Baseline	−3 ± 10.8	−4 ± 7.5			
Week 5	−1.7 ± 9.7	−1.3 ± 9.8	1.27	−0.91 to 3.4	.253
Week 9	−0.1 ± 9.9	0.8 ± 10.7	2.90	0.72 to 5.09	.009
Week 13	0.1 ± 9.8	0.9 ± 10.5	3.09	0.91 to 5.27	.006

*Note*: All data are shown as mean ± standard deviation. No significant effect for age (included as covariate in the model).

Abbreviations: 1‐MSTS, 1‐minute sit to stand test; 3‐MSTEP, 3‐minute step test; AUD, audio‐guided training; SR, sit and reach test; SUP, supervised training; TUG‐LV, Timed Up and Go test‐low vision version.

Table 3 shows the estimated MCIDs and the actual differences during and at the end of the training programs. At the end of training, the actual differences were higher than MCIDs for almost all tests apart from the TUG‐LV for SUP (equal difference as MCID) and SR for AUD (lower difference than MCID).

**TABLE 3:** Minimal clinically important differences for physical tests.

	Group	MCID	Δ5 wk	Δ9 wk	Δ13 wk	Posttraining
TUG‐LV (s)	AUD	1.6	−2.8	−3.1	−3.4	↑
	SUP	2.9	−1.8	−2.6	−2.9	=
1‐MSTS (reps)	AUD	3.1	4.1	6	5.8	↑
	SUP	3.6	7.8	8	11.2	↑
3‐MSTEP (reps)	AUD	8.5	11	13.2	16.8	↑
	SUP	10.8	19.7	26.7	30.2	↑
SR (cm)	AUD	5.4	1.3	2.9	3.1	↓
	SUP	3.7	2.7	4.8	4.9	↑

*Note*: MCIDs were estimated using the distribution‐based method at baseline. ↑ = difference post training > MCID; “=” = difference post training = MCID; ↓ = difference post training < MCID.

Abbreviations: 1‐MSTS, 1‐minute sit to stand test; 3‐MSTEP, 3‐minute step test; AUD, audio‐guided training; MCID, minimal clinically important differences; SR, sit and reach test; SUP, supervised training; TUG‐LV, Timed Up and Go test‐low vision version.

The level of physical activity increased due to the introduction of the exercise program (*p* < .001), without significant time per group interaction. At the baseline, the AUD group reported a physical activity level of 363 ± 520 MET‐min/week, which increased to 969 ± 808 MET‐min/week at the end of the exercise protocol. Similarly, the SUP group reported a physical activity of 612 ± 522 MET‐min/week at the baseline, and 1419 ± 526 MET‐min/week at the end of the exercise protocol.

### Clinical scales

In analyzing the participants' quality of life, we examined their satisfaction levels in the psychological and physical domains, as well as the health and social aspects related to visual functioning.

Despite improvements in motor function and physical activity levels, the satisfaction profile in both groups did not change significantly. The increase in psychological functioning was not statistically significant by the end of the exercise protocol, and no difference was observed between the groups.

The satisfaction on psychological functioning increased from 64.7 ± 16.6 to 66.9 ± 13.6 for the AUD group and from 69.8 ± 6.7 to 74.5 ± 8.6 for the SUP group. The physical functioning in the AUD group changed from 70.2 ± 15.8 to 73.6 ± 10.6 and in the SUP group from 67.9 ± 14.4 to 75.5 ± 12.8.

Perceived general health related to visual functioning did not change after completing the training, and there was no interaction effect between the groups. General health scores increased from 44.4 ± 16.7 to 50 ± 13.4 in the AUD group, and from 56.3 ± 29.1 to 65.6 ± 29.7 in the SUP group. The VFQ‐25 indicated no significant changes in scoring regarding completion of near activities. The scores varied from 26.9 ± 13 to 21.9 ± 16.6 in the AUD group and from 45.8 ± 10.9 to 54.2 ± 10.9 in the SUP group.

The social functioning improved over time, but changes are not statistically significant, with no difference between the groups. Data changed from 22.9 ± 13.5 to 34.4 ± 24.8 in the AUD group and from 48.4 ± 23.6 to 64.1 ± 18.2 in the SUP group.

The two modalities of training seem to have no effect on mental health, which remained unchanged over time (from 59 ± 16.9 to 58.6 ± 20.8 in AUD; from 38.3 ± 18.7 to 46.1 ± 22.1 in SUP group) and between groups.

Similarly, the role difficulties due to the visual impairment did not change over time, but there was a significant difference between groups (*p* = .009). Data varied from 72.2 ± 23.3 to 64.1 ± 25.4 in AUD, and from 29.7 ± 24.9 to 25 ± 31.3 in SUP group.

Finally, the level of dependency did not change over time, and in relation to the group. The mean dependency score for AUD group decreased from 75 ± 21.7 to 59.4 ± 30.4, and for SUP group remained almost unchanged, from 41.7 ± 30.5 to 42.7 ± 26.1.

The total score of the Caregiver Burden Inventory showed no significant changes after the training in either group. Scores slightly decreased from 16.3 ± 13.6 to 14.8 ± 15.2 in the AUD group and from 9.1 ± 13.5 to 5.9 ± 6.2 in the SUP group.

## DISCUSSION

People who are blind or have low vision often engage in insufficient physical activity, which can negatively impact on their mobility and overall quality of life. Previous studies have shown that certain forms of exercise, such as tai chi, yoga, and dance, can improve mobility, health, and well‐being in visually impaired individuals.^25^ However, many visually impaired people face challenges in leaving their homes and to participate in activities at gyms or outdoor places. In these cases, promoting physical activity at home could offer great benefits, but currently there is limited evidence on the effectiveness of self‐administered home training programs for people with low vision.

In our study, we assessed the feasibility and effectiveness of a bodyweight, home‐based and self‐managed audio‐guided exercise protocol for visually impaired individuals. As no injuries or physical issues were reported –aside from one participant who discontinued for personal reasons‐ and with an adherence rate >95% to the workouts, we conclude that the audio‐guided training is feasible for home use.

As previous studies demonstrated, supervised training programs are effective in improving strength and functional capacities in this population.^8^, ^25^ While most of the interventions in these studies were focused on lower limb strengthening, balance, or mind–body practices (yoga and tai chi)—with the aim of preventing falls in older adults—our exercise protocol was designed specifically to implement a structured concurrent (resistance and aerobic) training for people with low vision or blind at home. As in previous studies, we found improvements in functional capacities and exercise capacities (measured as TUG and chair STS tests). Similar to the supervised training, the audio‐guided protocol led to improvements in physical tests, suggesting it can be a suitable self‐administered alternative to supervised training. Posttraining differences in physical performance exceeded the majority of baseline MCID thresholds in both groups, confirming that each group derived clinically meaningful benefits from the training program.

However, although most participants improved with the intervention, a small number of tests exhibited a worsening of their scores (see Figure 1). This variability is consistent with previous findings on interindividual responses to exercise or health interventions, which report the existence of “nonresponders” or even “adverse responders” in some cases.^26^, ^27^ Possible reasons may include different baseline fitness, genetics, external stressors, or measurement error. These findings further highlight the need for personalized approaches in intervention design. At the onset and at the end of the exercise programs, we observed an increase in physical activity levels in both groups—from baseline low levels to a moderate level^28^—likely attributable to their involvement in the training. Previous research has highlighted the importance of physical activity for enhancing the health‐related quality of life in participants with visual impairments.^25^, ^29^, ^30^ In the present study, we assessed participants' satisfaction levels in psychological and physical domains, as well as in health and social aspects related to visual functioning. Although satisfaction levels improved after completing the training, only the physical domain showed a significant change. No significant changes were found in mental health, role difficulties, or dependency on others.

Given these findings, we can conclude that audio‐guided training can effectively help people with visual impairment improve their motor function. Although supervised training remains preferable due to the comparable or greater improvements in most evaluations, audio‐guided training offers a valuable alternative when supervision is not feasible.

Several limitations must be considered to ensure an accurate interpretation of the data. First, the unbalanced group sizes within the small sample, with only 19 participants completing the intervention, may limit both the statistical power and the generalizability of the findings. We cannot exclude the possibility of allocation bias, as group assignment was influenced by participants' ability to access transportation to the gym. Additionally, the 12‐week intervention lacked long‐term follow‐up assessments, making it difficult to assess the sustainability of the observed benefits. The data about physical activity level were self‐reported, so vulnerable to over‐ or underestimation by the participants themselves. The significant age difference and visual impairments among participants (eg, blindness or low acuity, central or peripheral visual field loss, congenital or acquired) could also have influenced the findings. Further research is warranted to stratify participants by age, the underlying etiology, and duration of visual impairment to gain a more nuanced understanding of how these variables modulate the impact of exercise interventions. Lastly, although physical performance improved, no significant changes were observed in several quality‐of‐life measures, such as mental health and dependency, suggesting the intervention's limited impact on broader aspects of daily life.

## CONCLUSION

The self‐administered, audio‐guided training performed in this study showed feasibility among people with severe visual impairments. Moreover, it effectively enhanced mobility and motor function in participants. Although supervised training should be preferred in improving mobility in this population due to its higher efficacy, audio‐guided training offers a valuable alternative in situations where supervision is not viable.

## DISCLOSURE

The authors report there are no competing interests to declare.

## ETHICS STATEMENT

The present study was approved by the local Ethical Committee (protocol number: 2422) and it is in accordance with the Declaration of Helsinki.

## PATIENT CONSENT STATEMENT

Before to be enrolled in the present study, each participant signed—at the presence of his/her caregiver or legal tutor—a detailed informed consent.

## Supporting information


**Data S1.** Supporting Information.

## Data Availability

The raw data supporting the conclusions of this article will be made available by the authors, without undue reservation.
