# Supplementary material for: Audio‐guided exercise at home: Preliminary findings from a pilot study on physical activity for people with low vision
Source: PM R. 2026 Jan 10;18(8):902–9. doi: 10.1002/pmrj.70081 (PMC13476666; doi:10.1002/pmrj.70081)
Supplement: Supplementary file 1 [file PMRJ-18-902-s001.docx]

**SUPPLEMENT A**

The exercise program consisted of two weekly resistance training sessions, with two rest days in between, for a total duration of twelve weeks. Every four weeks, the program was modified by increasing the training volume (repetitions and/or time; see the Table). The training volume for repetition and timed exercises was raised by 20% between the first and second month.

|  |  | Series x n° of repetitions or seconds | | |
| --- | --- | --- | --- | --- |
| *Phase* | *Exercise* | *Month 1* | *Month 2* | *Month 3* |
| *Proprioception* | 1. Load Shifting | 1x20 | 1x24 | 1x30 |
|  | 2. Calf raise | 1x15 | 1x18 | 1x22 |
|  | 3. Toe to Heel | 1x10 | 1x12 | 1x14 |
| *Warm-up* | 4. Arms circumductions | 1x20 | 1x24 | 1x30 |
|  | 2. Calf raise | 1x20 | 1x24 | 1x30 |
|  | 3. Toe to Heel | 1x20 | 1x24 | 1x30 |
| Strength phase | 7. Squats | 2x10 | 2x12 | 2x14 |
|  | 8. Isometric squats on the wall | 2x30" | 2x36" | 2x43" |
|  | 9. Lunges (per leg) | 2x10 | 2x12 | 2x14 |
|  | 10. Push-ups | 2x10 | 2x12 | 2x14 |
|  | 11. Rowing with elastic band | 2x10 | 2x12 | 2x14 |
|  | 12. Lateral abductions (e. band) | 2x10 | 2x12 | 2x14 |
| Aerobic phase | 13. Walk on the spot | 4x1' (15"Rest) | 5x1' (15"Rest) | 6x1' (15" Rest) |
| Cool-down | 14. Stretching for hamstrings | 2x10 | 2x12 | 2x14 |
|  | 15. Stretching for upper body | 2x30" | 2x36" | 2x43" |
|  |  |  | *volume +20%* | *volume +40% (compared to month 1)* |
